# Occurrence and trajectories of psychiatric disorders in adults with gambling disorder: a longitudinal register-based study of the Finnish population

**DOI:** 10.1007/s00127-025-03034-8

**Published:** 2026-02-13

**Authors:** Jussi Palomäki, Tanja Grönroos, Maria Heiskanen, Jonna Levola, Anne H. Salonen

**Affiliations:** 1 Department of Promotional and Preventive Work, Finnish Institute for Health and Welfare, P.O. Box 30, Helsinki, FI-00271 Finland; 2 Department of Digital Humanities, University of Helsinki Cognitive Science, Helsinki, Finland; 3 Faculty of Information Technology and Communication Sciences, Tampere University, Tampere, Finland; 4 Faculty of Social Sciences, University of Helsinki, P.O. Box 54, Helsinki, FI-00014 Finland; 5 Department of Psychiatry, University of Helsinki and Helsinki University Hospital, P.O. Box 22, Helsinki, FI-00014 Finland; 6 Faculty of Health Sciences, University of Eastern Finland, Kuopio, Finland

**Keywords:** Gambling disorder, Comorbidity, Mental disorders, Psychiatric disorders, Register study, Disease trajectories, Epidemiology

## Abstract

**Purpose:**

To determine the co-occurrence and temporal trajectories between gambling disorder (GD) and other psychiatric diagnoses in the Finnish population.

**Methods:**

Data were retrieved from two healthcare registers covering primary and specialized care, including 5,172 individuals aged 18 or over (3,720 males, 1,452 females) with diagnosed GD (ICD-10 code F63.0). We also retrieved information on all other psychiatric diagnosis dates (codes F00–99) between 1996 and 2024 for every individual, alongside their official sex, year of birth, and whether GD was a primary, secondary, or long-term diagnosis. Linear mixed modelling (LMM) was used to analyse longitudinal data on multiple diagnosis occurrences.

**Results:**

Mood, anxiety, and substance use disorders (SUDs) had the highest co-occurrence with GD both preceding and following its diagnosis, indicating bidirectional comorbidity patterns. The most common comorbid categories included unipolar depression, phobic anxiety disorders, alcohol use disorder, and bipolar disorder. Mood disorder comorbidity was especially prominent among women, and SUD comorbidity among men. Most psychiatric diagnoses occurred 112 to 2,697 days before GD (LMM *p*-values 0.38 – <0.001, marginal and conditional R² = 0.13 and 0.47, respectively). Comorbid diagnoses after GD were, on average, temporally closer to GD onset than diagnoses before GD.Diagnosis occurrences were significantly more frequent among individuals with GD than among psychiatric controls in most diagnostic categories.

**Conclusions:**

There were distinct and sex-specific temporal trajectories between GD and other psychiatric diagnoses, with mood-, anxiety-, and SUDs showing the strongest and most reciprocal associations. Social and health care professionals should screen for GD among those seeking treatment for these disorders, particularly given the increased suicide risk.

**Supplementary Information:**

The online version contains supplementary material available at https://doi.org/10.1007/s00127-025-03034-8.

## Introduction

In 2023, 70% of Finns aged 15 to 74 reported having gambled in the past year [1]. Gambling is a popular pastime, but for some individuals, it can have serious harmful consequences. The negative consequences of gambling, such as financial hardship, emotional distress and even suicide, are a serious public health concern affecting gamblers, families, workplaces, and communities [2].

Gambling disorder (GD) is defined as a behavioural pattern of excessive gambling with severe negative consequences and loss of control according to both the Diagnostic and Statistical Manual of Mental Disorders (5th ed.; DSM-5) [3] and the World Health Organization’s International Classification of Diseases (11th revision; ICD-11) [4]. GD is a behavioural addiction with similar risk factors and symptoms to those of substance use disorders (SUDs), including impaired control, persistent engagement despite negative consequences, and craving-like experiences [5, 6]. These parallels extend to neurobiological mechanisms and comorbidity patterns, supporting the classification of GD within the addiction spectrum. GD is more prevalent in men than women [7, 8], although the prevalence among women is increasing in Finland and Sweden [9, 10]. Women typically start gambling at an older age but develop problem gambling and seek treatment for it sooner than men [11, 12]. Women are also more likely than men to gamble as a coping strategy for depression and anxiety; however, problem gambling can exacerbate these symptoms [13].

Epidemiological studies on problem gambling have largely relied on self-report survey methodology [1, 14]– [15], which has known limitations such as participants’ self-selection into the surveys and several social biases influencing their responses. The prevalence of problem gambling has ranged from 0.5% to 3.3% in Finland depending on the time period and survey instruments used [16]. While problem gambling can be detected using self-report measures, in Finland, a formal GD diagnosis can only be set by a physician based on an evaluation of an individual fulfilling diagnostic criteria. The survey-based population estimates of GD are significantly higher than the number of GD diagnoses assigned by physicians, suggesting underdiagnosis of the disorder [9]. Similar work in alcohol research has differentiated survey- and register data-based prevalence estimates [17]. Addictive disorders can also present within the scope of social-, rather than health care services, where physicians may not as often be consulted.

The Finnish public services are obligated to organise affordable health- and social care, and the healthcare system is divided into primary- and specialized healthcare in regional- and university hospitals [18]. Accessing specialized healthcare requires a physician’s referral. Many people without a formal GD diagnosis receive interventions, such as online self-help programs or brief interventions from health and social care professionals who are not physicians. On the other hand, many individuals wait for several years to seek treatment for their existing gambling problem due to unwillingness to admit gambling, inability to recognize gambling as a problem, wanting to handle their problems alone, feelings of shame, or fear of stigma [19]– [20]. Individuals may also not recognize when GD underlies comorbid psychiatric symptoms, such as depression, anxiety or sleep problems, and it is common for the individual to seek treatment for something else than GD itself. Individuals with diagnosed psychiatric disorders likely use health care services more frequently than undiagnosed individuals, and thus it would be crucial for successful treatment of these to screen also for potential GD and treat it accordingly.

National health register data allow for examining the administrative prevalence rate of GD and its comorbidities while avoiding many of the pitfalls in self-report surveys [21, 22]. Grönroos and colleagues [21] found that comorbid psychiatric diagnoses were significantly more common than somatic illnesses in individuals with diagnosed GD. Of these individuals, 87% had at least one comorbid psychiatric disorder diagnosis. Similarly, Håkansson and colleagues [10] found that mood-, anxiety-, and SUDs were the most prevalent comorbid conditions of GD, with mood and anxiety disorders being more prevalent in women than men. Women were also more likely than men to receive comorbid psychiatric diagnoses before GD, while men were more likely to receive the diagnoses simultaneously [22]. Moreover, Sundqvist and colleagues [23] found that comorbid conditions such as anxiety, depression and problems with substances were more typically observed before developing problem gambling among women, but after developing problem gambling among men. Girard and colleagues [24], in turn, observed a directional path from SUD to GD among both sexes without support for the reversed path. However, while the co-occurrence between GD, anxiety, depression, SUD, and personality disorders is clear, their causal pathways have not been conclusively established [25].

This study examines decades of individual-level register data to determine the temporal order between GD and other psychiatric diagnoses. Although register-based data primarily reflect healthcare service use and physician-assigned diagnoses rather than true population prevalence [17], they provide essential insights into prior diagnoses, timelines of comorbid psychiatric disorders, and opportunities to improve the recognition of GD in healthcare [26]– [27]. We address three research questions:

RQ 1) What is the overall occurrence of comorbid psychiatric disorders of GD?

RQ 2) To what degree do comorbid psychiatric disorders precede or follow the GD diagnosis?

RQ 3) What are the individual-level temporal trajectories between diagnosed GD and other comorbid psychiatric disorders?

## Methods

The STROBE checklist was used to report the methods and results of the study (table S3) [28]. The study was not pre-registered.

### Participants, variables, and procedures

We obtained individual-level data on GD diagnoses (ICD-10 code: F63.0^1^) for individuals aged 18 or older at the time of their first primary, secondary or long-term^2^ GD diagnosis from two national registers administered by the Finnish Institute for Health and Welfare covering the entire Finnish population: (1) Register of Primary Health Care (RPHC), covering visits to primary healthcare centers, occupational healthcare services, and private clinics (1996–2024); and (2) the Care Register for Health Care (CRHC), covering specialized outpatient and inpatient healthcare as well as inpatient social care services^3^ (2011–2024). The registers include medical diagnoses and their registry dates. The final sample comprised of 5,172 individuals (3,720 males, 1,452 females) diagnosed with GD (Table 1). We also obtained information on the individuals’ comorbid psychiatric disorders (ICD-10 categories F00–99) diagnosed before, after, or at the same time as GD. Individuals may appear in both registers, but a single visit is recorded in either RPHC or CRHC, not both. Thus, individuals in our data have as many rows of data as they have observations in both RPHC or CRHC, with each row linked to only one register.

**Table 1:** Data summary

	Sex		Health register		Total
	Male	Female	RPHC	CRHC	
Number of individuals	3,720	1,452	2,621	2,551	5,172
Number of observations	28,653	14,542	27,404	15,791	43,195
Average year of birth (SD)	1,982.9 (13.9)	1,975.8 (16.4)	1,977.4 (15.9)	1,984.5 (13)	1,980.9 (15)
Year of birth range	1920–2006	1912–2005	1912–2006	1924–2006	1912–2006
Average age at first GD diagnosis (SD)	34 (11.5)	40.4 (13.9)	36.1 (12.9)	35.5 (12.3)	35.8 (12.6)
Age at first GD diagnosis range	18–88	18–87	18–86	18–88	18–88
Average number of F-diagnoses (SD)	7.7 (6.86)	10.02 (7.77)	10.34 (7.79)	6.31 (5.88)	8.32 (7.2)
Number of F-diagnoses range	1–61	1–52	1–56	1–61	1–61
% GD as primary diagnosis	36.21	29.27	31.3	37.3	34.3
% GD as long-term diagnosis	2.72	3.86	4.27	1.76	3.04

Note. RPHC = Register of Primary Health Care (1996–2024); CRHC = Care Register for Health Care (2011–2024); GD = Gambling Disorder; SD = Standard Deviation

Based on the diagnosis date of entry, we further calculated, for every individual, a variable indexing the *Difference in days* (number of days ranging from − 10,446 to 10,420; expressed as years in visualizations for clarity) between having been given the GD diagnosis and any other F-diagnosis. The data were organized into long format with multiple rows per individual. Each row corresponds to an F-diagnosis given at a specific date, such that one diagnosis appears once for each individual (based on the date it was first given). In other words, each data row is a single observation corresponding to a recorded first diagnosis during a health-care visit to a physician. For analysis, comorbid disorders were grouped into 11 ICD-10 categories: organic mental disorders (F00–09), SUD (F10–19), schizophrenia spectrum disorders (F20–29), mood disorders (F30–39), anxiety disorders (F40–48), behavioural syndromes associated with psychological disturbances and physical factors (F50–59), personality disorders (F60–69, excluding F63.0), intellectual disability (F70–79), disorders of psychological development (F80–89), behavioural and emotional disorders in childhood and adolescence (F90–98), and unspecified mental disorders (F99).

### Statistical analysis

We used the R platform for statistical computing (v. 4.4.2) [29], and linear mixed modelling (LMM) within the lme4 package [30]. There were multiple observations from individuals, each observation corresponding to a distinct F-diagnosis given. Thus, individual ID was used as a level 2 clustering variable and a random intercept effect, meaning variance was allowed in the intercepts between individuals. Although the number of observations per individual varied, LMM using maximum likelihood estimation can accommodate unequal numbers of observations in the dependent variable. *Difference in days* served as the continuous dependent variable. *ICD category* (11 levels; see above), *Sex* (male/female), *Register source* (RPHC/CRHC), and *GD diagnosis status* (primary/secondary diagnosis) were used as categorical predictors. We also modelled the interaction between *ICD category* and *Sex*. The *ICD category* level “gambling disorder” was used as the reference level^4^.

The lmerTest package [31], which applies Satterthwaite’s method, was used to estimate degrees of freedom and *p-*values. Marginal and conditional *R*^2^ values [32] were obtained, which estimate the variance explained by fixed factors and fixed plus random factors, respectively. The LMM satisfied the linearity assumption. Q-Q plots indicated that the model residuals and random effects were somewhat normally distributed and homoscedastic. Posterior predictive checks [33] showed the LMM predicted fewer zero-values than observed. Robustness checks using zero-inflated Gaussian mixed modelling [34], robust LMM [35], and Bayesian LMM [36] produced similar results; only initial LMM results are reported.

To evaluate temporal trajectories of F-diagnoses, we created Sankey diagrams visualizing participants’ flow from their first to tenth diagnosis (further diagnoses were omitted for clarity). In the diagrams, vertical column height represents the number of individuals, colors indicate F-diagnoses, and wavy columns represent transitions between diagnoses. As the diagnosis number increases, the vertical column height decreases because fewer individuals have received increasingly many diagnoses. Individuals flow from one F-category into the same F-category if their subsequently given diagnosis belongs to the same category. The GD diagnosis is distinct and can only flow into another F-category.

## Results

### GD and comorbid psychiatric disorders (RQ 1)

Comorbidity between GD and a given F-diagnosis category is represented by diagnosis occurrence counts in each category. GD was most strongly associated with mood disorder diagnoses (13,493 observations in total), followed by anxiety disorders (8,518 observations), SUD (6,635 observations), schizophrenia spectrum disorders (2,619 observations), personality disorders (2,140 observations), behavioural syndromes (1,879 observations), behavioural and emotional disorders in childhood and adolescence (1,440 observations), disorders of psychological development (600 observations), organic mental disorders (299 observations), unspecified mental disorders (288 observations), and intellectual disability (112 observations). Note that counts may exceed the number of individuals, as one person can receive multiple diagnoses within a category (see Table S1 for disaggregated counts).

As a supplementary analysis, we compared diagnosis occurrence counts for individuals with GD against a birth-year- and sex-matched control group comprising individuals with at least one psychiatric diagnosis other than GD (“psychiatric controls”). Psychiatric controls were aged 18 or older and randomly sampled from the RPHC and CRHC registries such that sample size, sex distribution, and sex-wise average birth years (and SDs) matched the GD group, for the same data periods (1996–2025 for RPHC and 2011–2025 for CRHC^5^).

We calculated the number of diagnoses (excluding GD) across all ICD-10 F-categories for both groups; detailed counts are reported in the Supplementary Materials (see Table S2). GD counts represent population parameters; thus, statistical inference is applied only to psychiatric controls. For the controls, we used cluster bootstrapping – resampling participants with replacement (1,000 iterations) – to obtain 99% confidence intervals (CIs) for diagnosis occurrence counts (see Table S2). When the GD population parameter lay outside these intervals, we interpreted the difference as statistically significant (~ 0.01 level).

Compared with the psychiatric controls, the GD group had significantly (*p* <.01) more diagnosis occurrences in almost all categories, notably in mood, anxiety, substance use, schizophrenia spectrum, attention deficit hyperactivity disorder (ADHD) spectrum, and personality disorders. Psychiatric controls had significantly higher diagnosis counts for somatoform disorders, sexual dysfunction, and intellectual disability (overall counts were low). Overall, individuals with GD had substantially more registered psychiatric diagnoses than the matched controls.

### Temporal order of GD diagnosis and its comorbid disorders (RQ 2)

**Fig. 1:**
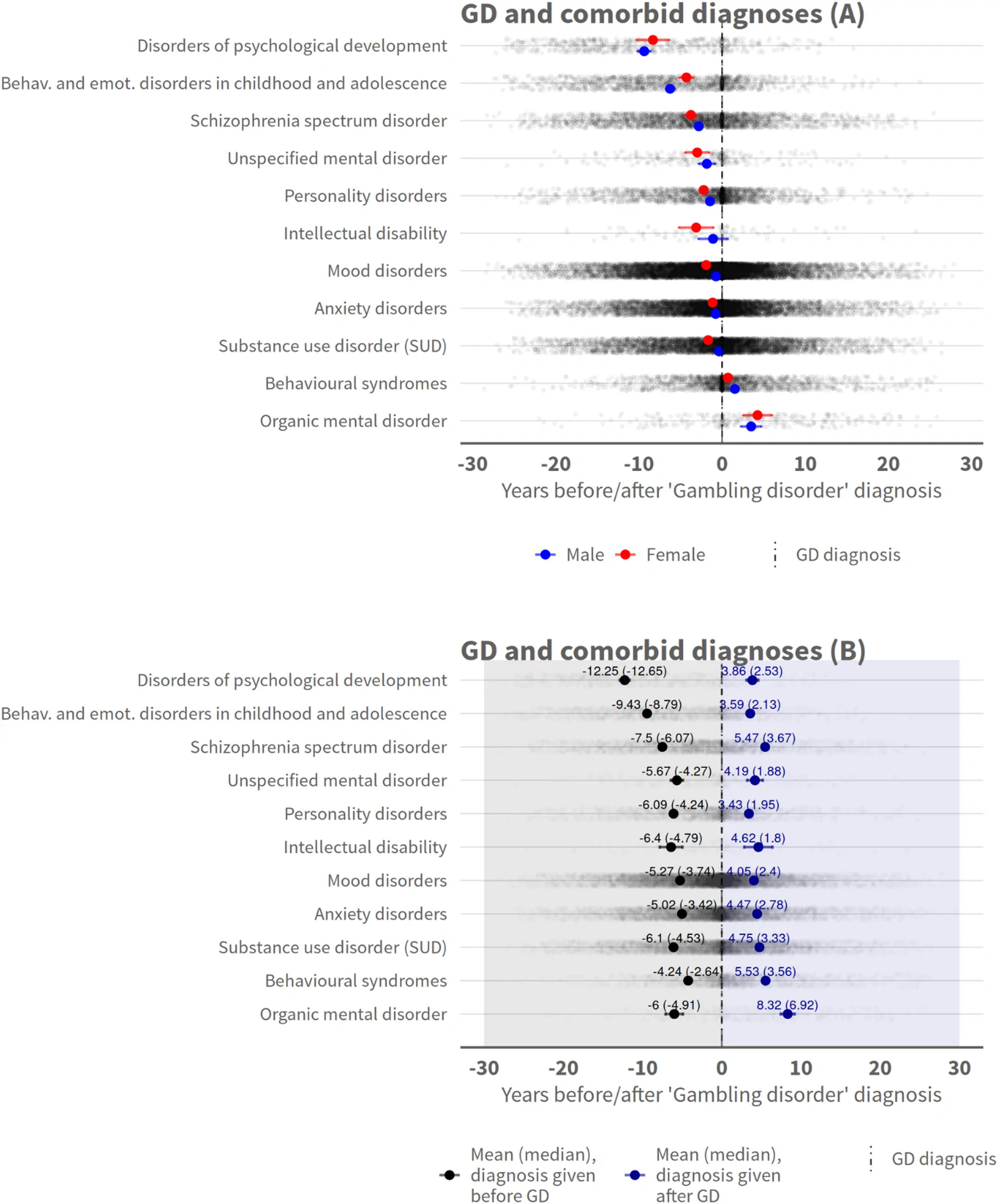
(**A** and **B**). Difference in time between receiving a gambling disorder (GD) diagnosis and a comorbid psychiatric diagnosis. Black/grey data points represent individual observations of the onsets of different comorbid diagnoses. Random vertical jitter is added to data points. **A **(top panel): The means for male (blue points) and female (red points) calculated from raw data. **B** (bottom panel): The means (and medians) calculated separately for values above (dark blue) and below (black) zero, with zero-values excluded from the data. The error bars are 95% confidence intervals

Figure 1A shows the mean difference-in-days values (expressed in years before or after the GD diagnosis) for all F-diagnosis categories and both sexes, across the full range of observations. Figure 1B presents separate mean *Difference in days* -values for comorbid diagnoses given *before* or *after* GD, excluding zero-values (diagnoses given on the same date as GD). Table 2 presents the LMM estimates. Sex-wise means in Fig. 1A are calculated from raw data, so they differ slightly from the LMM predictions. In the Supplementary Materials, the results are visualized separately for primary vs. secondary- (figure S1), long-term vs. non-long-term GD diagnosis status (figure S2), the two data source registers (figure S3), restricting the data range to years 2011–2024 for both registers as a robustness check and to enable full comparability between the registers (figure S4), and for all disaggregated specific diagnoses (figure S5). Notably, the disaggregated analysis (figure S5) further shows that alcohol use-, opioid use-, cannabis use-, and cocaine/other stimulant use disorders are diagnosed on average slightly before GD, particularly for females; while tobacco use disorder is diagnosed, on average, slightly after GD. Eating disorders are diagnosed before GD for both sexes, while nonorganic sleeping disorders are diagnosed, on average, after GD. Moreover, ADHD is diagnosed, on average, before GD for males, but around the same time as GD for females.

Behavioral syndromes (mainly non-organic sleep disorders) and organic mental disorders were typically diagnosed *after* GD, whereas all other F-diagnoses occurred, on average, *before* GD (Fig. 1A and B). In the LMM analysis, the largest effects were observed for the ICD categories of disorders of psychological development (B = −2,679.9, SE = 88.9, t = −30.13, *p* <.001), and behavioural and emotional disorders in childhood and adolescence (B = −1,684.1, SE = 63.68, t = −26.45, *p* <.001), which were predicted to be diagnosed 2,679 and 1,684 days before GD, respectively. Personality disorders (B = −337.16, SE = 61.02, t = −5.53, *p <*.001), anxiety disorders (B = −267.74, SE = −6.94, t = −6.94, *p* <.001), schizophrenia spectrum disorders (B = −243.14, SE = 55.22, t = −4.4, *p* <.001), and mood disorders (B = −199.82, SE = 36.02, t = −5.55, *p <*.001) were predicted to be diagnosed 337, 267, 243 and 199 days before GD, respectively. SUD (B = 166.99, SE = 41.49, t = 4.03, *p* <.001) and organic mental disorders (B = 1128.62, SE = 139.02, t = 8.12, *p* <.001) were predicted to be diagnosed 167 and 1,128 days after GD, respectively. Register source also had a significant effect. The CRHC register begins in 2011, and RPHC in 1996; therefore, *Difference in days* values tend to be higher for the CRHC register.

A significant interaction between ICD category and sex indicated that *Difference in days* values were lower for females than males when the comorbid diagnosis was in mood disorders, schizophrenia spectrum disorders, behavioral syndromes, personality disorders, or SUDs; but higher for females than males if the diagnosis given belonged in disorders of psychological development, or behavioural and emotional disorders in childhood and adolescence (cf. Fig. 1A; Table 2). In some of the robustness check analyses these interactions were not as prominent and should therefore be interpreted with caution. Age at GD diagnosis entered as a time-invariant control had no meaningful effect.

**Table 2:** LMM parameter estimates for fixed effects (in increasing B-value order), random effects and model fit

DV = Difference in days	B-value	SE	t-value	df	*p*-value
**Intercept**	−690.25	48.62	−14.20	12,255.79	< 0.001
**ICD category (ref: Gambling disorder)**					
Disorders of psychological development	−2679.90	88.96	−30.13	41,500.47	< 0.001
Behav. and emot. disorders in childhood and adolescence	−1684.19	63.68	−26.45	41,424.61	< 0.001
Personality disorders	−337.16	61.02	−5.53	40,561.92	< 0.001
Intellectual disability	−300.67	213.21	−1.41	41,466.72	0.16
Anxiety disorders	−267.74	38.56	−6.94	41,252.52	< 0.001
Schizophrenia spectrum disorder	−243.14	55.22	−4.40	42,246.13	< 0.001
Mood disorders	−199.82	36.02	−5.55	41,485.83	< 0.001
Unspecified mental disorders	−112.52	129.47	−0.87	40,410.48	0.38
Behavioural syndromes	105.99	59.29	1.79	41,067.89	0.07
Substance use disorder (SUD)	166.99	41.49	4.03	41,675.39	< 0.001
Organic mental disorders	1128.62	139.02	8.12	42,080.03	< 0.001
**Sex (ref: Male)**					
Female	117.41	67.85	1.73	19,097.43	0.08
**GD diagnosis status** **(ref: GD primary diagnosis)**					
GD secondary diagnosis	−65.79	47.78	−1.38	6,070.97	0.17
**Register source (ref: RPHC)**					
CRHC	1420.3	21.45	66.22	43,036.69	< 0.001
ICD category * Sex					
Intellectual disability * Female	−708.84	386.92	−1.83	40,704.75	0.07
Unspecified mental disorder * Female	−509.24	233.12	−2.18	40,057.47	0.03
Mood disorders * Female	−304.04	63.82	−4.76	41,062.00	< 0.001
Schizophrenia spectrum disorders * Female	−279.40	107.55	−2.60	41,987.79	0.01
Behavioural syndromes * Female	−268.48	102.85	−2.61	40,521.89	0.01
Personality disorders * Female	−252.87	96.10	−2.63	40,516.50	0.01
Substance use disorders (SUD) * Female	−218.43	78.42	−2.79	41,271.75	0.01
Anxiety disorders * Female	−123.89	67.96	−1.82	40,798.85	0.07
Organic mental disorders * Female	100.15	235.05	0.43	41,987.48	0.67
Behav. and emot. disorders in childhood and adolescence * Female	568.22	124.52	4.56	40,577.06	< 0.001
Disorders of psychological development * Female	640.07	217.90	2.94	40,577.97	< 0.001
**Random effects**					
Individual ID intercept SD	1,369.9				
Residual SD	1,703.1				
**Model fit**					
Marginal R^2^	0.13				
Conditional R^2^	0.47				
ICC (5172 groups [individuals], 43195 observations [measurement occasions])	0.4				

Type-3 ANOVA with Satterthwaite’s method was used for approximating the degrees of freedom and calculating *p-*values. B-values refer to “Difference in days” of having been given a comorbid F-diagnosis compared with when GD was diagnosed (GD is the reference level and represents the “Difference in days” value of 0; this can also be interpreted as “Difference in years” by dividing the numbers by 365). Thus, for the ICD category predictors, negative values indicate that a specific diagnosis was given earlier than GD, while positive values indicate that a specific diagnosis was given later than GD. For the other predictors (Sex, GD diagnosis status, Register source), the B-values represent the average “Difference in days” value across all ICD category levels. For example, for the predictor “Female”, the B-value of 117.41 indicates that, on average across all ICD categories, females were given comorbid F-diagnoses 117.41 days later than males with respect to their GD diagnosis onset (though this difference is not statistically significant at *p* =.08). The interaction between ICD category and Sex indicates that for some specific ICD categories, females were given the comorbid F-diagnosis either *earlier* or *later* than males, with respect to their GD diagnosis onset (cf. the differences between the red and blue points in Fig. 1). Note also that while in the raw data there are only “0” values for the ICD category “Gambling disorder” (reference category), the intercept can be non-zero, and thus difficult to interpret, when there are other predictors in the model. Without other predictors besides ICD category, the model would have a fixed intercept of “0”

### Individual-level Temporal trajectories between diagnoses (RQ 3)

**Fig. 2:**
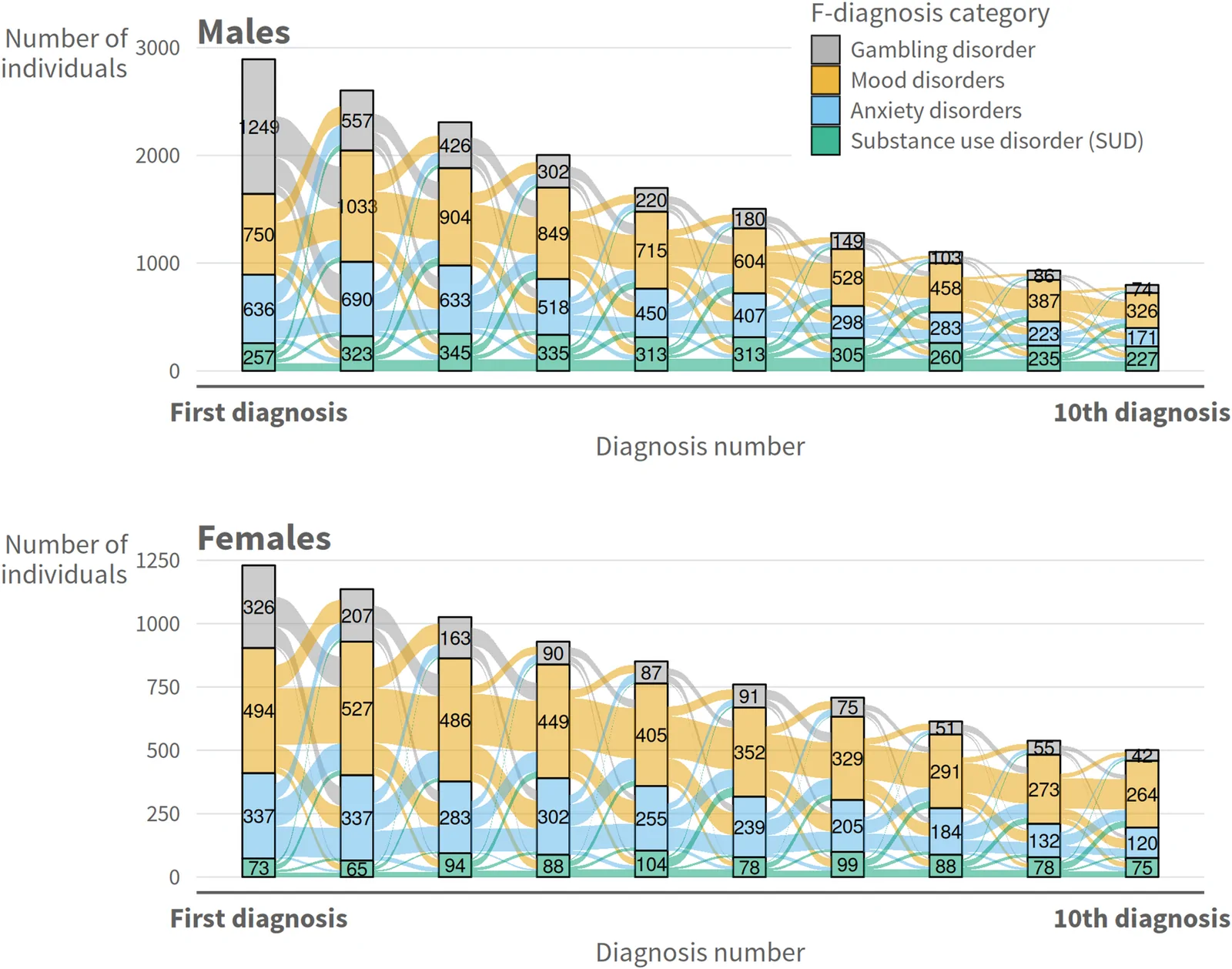
Temporal diagnosis trajectories between GD and the three most common comorbid psychiatric diagnosis categories. Data is excluded for diagnosis numbers above 10 for clarity

Figure 2 focuses on the three most common F-categories: mood, anxiety, and SUDs. Among individuals with GD as their first diagnosis (grey section, first bar), 30.6% of males and 35.9% of females were next diagnosed with mood disorders; 20.3% of males and 23.6% of females with anxiety disorders; and 7.0% of males and 2.8% of females with SUD (grey wavy lines; percentages not shown in the figure to reduce clutter). Among individuals with GD as their second diagnosis, 30.9% of males and 44.0% of females were previously diagnosed with mood disorders; 32.1% of males and 30.4% of females with anxiety disorders; and 10.6% of males and 5.8% of females with SUD. Similar patterns repeated across diagnosis numbers.

**Fig. 3:**
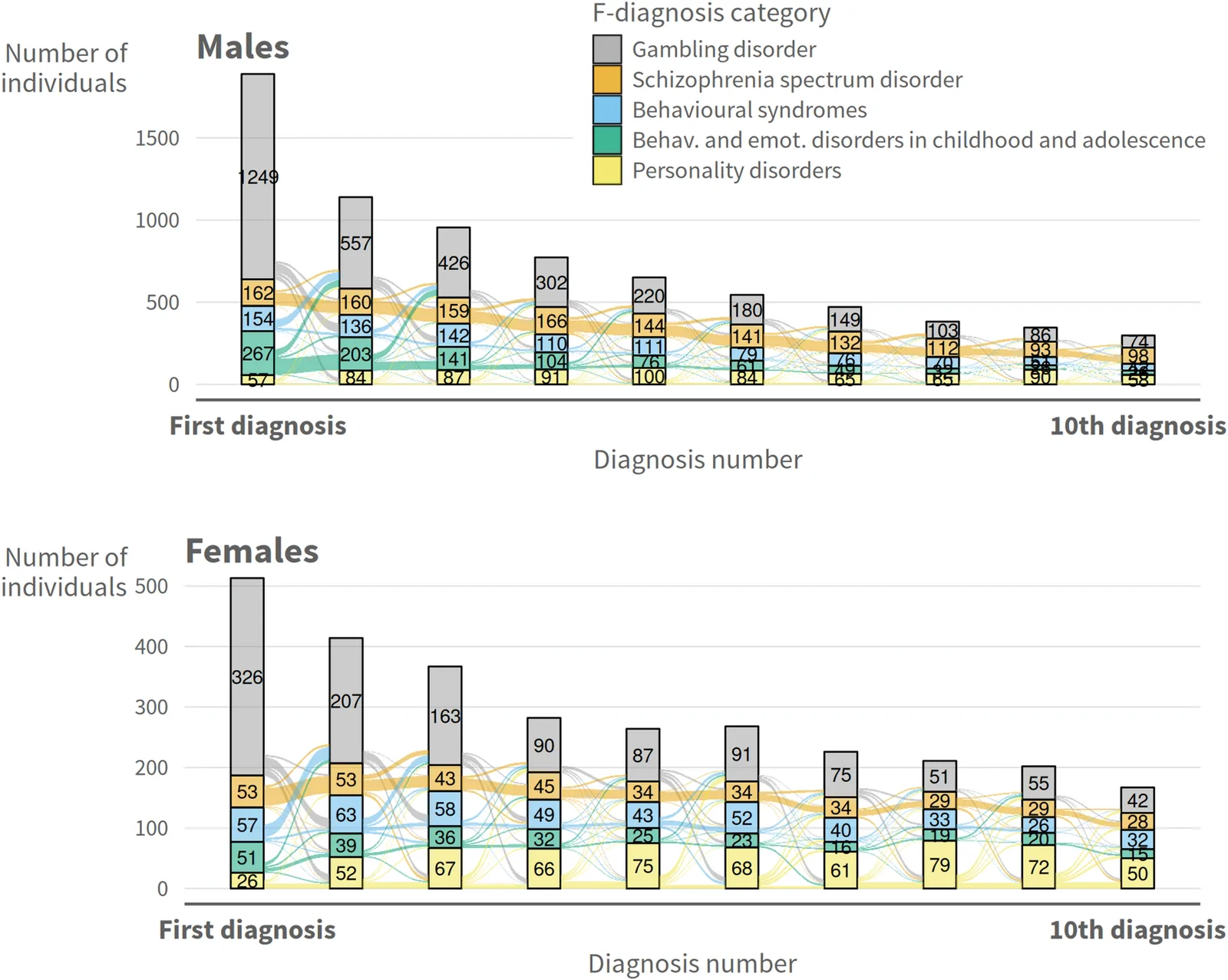
Temporal diagnosis trajectories between GD and the bottom 4–7 most common comorbid diagnosis categories. Data is excluded for diagnosis numbers above 10 for clarity

Figure 3 focuses on the 4–7 least common diagnosis-categories (alongside GD). A small subset of individuals first diagnosed with GD are subsequently diagnosed with schizophrenia spectrum disorders (males 1.0%; females 0.9%), behavioural syndromes (males 4.5%; females 4.9%), behavioural and emotional disorders in childhood and adolescence (males 2.0%; females 3.4%), or personality disorders (males 1.5%; females 1.2%). If GD was the second diagnosis, there was a minor chance that its preceding diagnosis belonged to schizophrenia spectrum (2.3% of males and 1.9% of females), behavioural syndromes (males 8.8%; females 10.1%), behavioural and emotional disorders in childhood and adolescence (males 7.4%; females 2.9%), or personality disorders (males 2.3%; females 0.05%). This pattern repeated across diagnosis numbers. Results for other F-categories are not visualized due to low counts.

## Discussion

We evaluated psychiatric diagnoses before and after GD using two national health registers covering both primary and specialized healthcare. GD was most strongly associated with mood, anxiety, and SUDs, particularly unipolar depression, phobic anxiety, adjustment and stress related disorders, and AUD, consistent with previous findings [37–39].

Mood, anxiety, and SUDs are generally the most prevalent diagnoses in psychiatric populations, and this pattern was also evident in our data [40]. However, comparison with a sex- and age-matched psychiatric control group showed that individuals with GD did not merely receive the most common psychiatric diagnoses by base-rate chance. Rather, diagnosis occurrences were more frequent among individuals with GD than among psychiatric controls in almost all categories, particularly mood, anxiety, and SUDs. These findings suggest that the comorbidity profile of GD reflects a broader and more intensive psychiatric burden, with disproportionate vulnerability to affective, anxiety-related, and substance-related conditions, rather than simply mirroring the diagnostic landscape of the general psychiatric population. However, higher comorbid diagnosis counts may also partly reflect challenges in accurately pinpointing the “actual” problem (i.e., GD) in clinical settings.

Most (69.6%) GD diagnoses followed another psychiatric diagnosis; for 30.4% of individuals (*n* = 1,572), GD was the first. Comorbid diagnoses in 8 of 11 categories were set before GD on average, although substantial variability existed in the *Difference in days* variable. This pattern is expected for disorders arising earlier in life, such as disorders of psychological development or behavioural and emotional disorders in childhood and adolescence, including ADHD spectrum disorders [41–43]. ADHD often manifests early, but in our data, this was mainly true for males—female ADHD diagnoses tended to occur around the same time as GD, suggesting under-recognition or delayed diagnosis among females (cf. figure S5). Diagnoses after GD were temporally closer to its onset than those occurring before it, indicating that receiving a GD diagnosis may influence subsequent diagnostic activity. However, temporal proximity is not evidence of causality, and shared or unmeasured etiological factors may contribute to both GD and comorbid disorders. Symptom onset may also precede formal diagnosis, and latency varies across disorders.

Psychiatric disorders preceding GD may also be diagnosed earlier due to social and emotional barriers delaying help-seeking [19, 44]. Routine screening for gambling during psychiatric or primary-care visits—especially for patients with mood, anxiety, or SUDs—is therefore warranted. Disaggregated analyses (figure S5) showed nuanced temporal patterns across substance-use categories: AUD and other SUDs were more often diagnosed *before* GD, whereas tobacco use disorder was diagnosed *after* GD. These differences suggest heterogeneous relationships between gambling and substance-use problems: alcohol and other substances may act as antecedent vulnerabilities via shared reward-system sensitivity or disinhibition, whereas tobacco problems tend to appear later, potentially emerging as gambling intensifies or as exposure- and lifestyle-related factors increase risk. Clinically, this highlights the need for early screening and intervention for alcohol and other substance problems, with continued monitoring of tobacco use following a GD diagnosis.

Behavioural syndromes (notably non-organic sleep disorders), organic mental disorders, and SUD (only in the LMM analysis) were the few categories diagnosed after GD. Organic mental disorders appear later in life, possibly explaining this. Impaired impulse control related to frontal lobe conditions may exacerbate problem gambling, and dopaminergic medication (e.g., for Parkinson’s) increases GD risk [45]. Disaggregated analyses (figure S5) reveal divergent timing patterns for eating versus sleep disorders: eating disorders were typically diagnosed *before* GD, suggesting antecedent vulnerability, whereas non-organic sleep disorders were diagnosed *after* GD. This may reflect sleep disruption as a consequence of gambling problems or delayed recognition once gambling behaviour becomes problematic, underscoring the importance of assessing both past and current behavioural syndromes in GD evaluation.

The diagnostic “flow” visualizations showed that individuals, especially females, who were first diagnosed with GD were subsequently most likely to receive diagnoses of mood or anxiety disorders. Conversely, males first diagnosed with GD were more likely to later receive a SUD diagnosis. The severity of problem gambling tends to increase financial stress—particularly among women [46]—and financial stress, in turn, is strongly associated with depression [47]. For individuals receiving GD as a *subsequent* diagnosis, the preceding diagnosis was most often in mood or anxiety disorders (particularly among females) or SUD (among males). These findings complement earlier work showing that females, more so than males, often gamble to cope with feelings of depression and anxiety, thereby exacerbating their already impaired emotional situation [17, 48], and that females are more likely than males to engage with healthcare services [49].

The higher frequency of GD being recorded as a primary diagnosis among males may reflect how diagnostic prioritization operates in clinical settings. Because only one primary diagnosis is typically assigned per care episode – usually the condition judged to require the most clinical attention – GD may be more often prioritized for males if it is perceived as the most salient treatment issue during the visit. Additionally, administrative incidence rates of other comorbid somatic and psychiatric disorders (i.e., clinically diagnosed and recorded cases in healthcare data) are generally higher among females than males [50], which may partly explain the lower likelihood of GD appearing as the primary diagnosis in female patients.

## Limitations and future research

Our data involves individuals who have met a physician. Thus, the data did not cover individuals with gambling problems without a formal diagnosis, for example, within social services. Engagement in health care services increases the likelihood of receiving any diagnoses. Nevertheless, people are treated for problem gambling more frequently than formal GD diagnoses are given, but on the other hand, diagnosed GD does not mean the individual has received or been offered treatment for their GD. While limited to healthcare service users, our data allows for a deep dive into the functioning of the healthcare system.

In future work, combining register-based data with self-report measures would shed much needed light on how many individuals with self-reported problem gambling have been diagnosed with GD or with something else. Under-diagnosing addictive disorders in healthcare is common in Finland [9, 16, 17] and may be due to many causes. If employees fall ill, they are usually entitled to paid sick leave or sickness allowance from the Social Insurance Institution in Finland, which also supports rehabilitation (e.g. rehabilitative psychotherapy). Future research should examine diagnostic practices related to GD in Finland, including potential influences of benefit eligibility on coding decisions, and explore care pathways using linked routine data, as demonstrated by Möckl and colleagues [51]. Because GD or other addictive disorders are not sufficient causes for these benefits and may even hinder eligibility, physicians might diagnose patients with depression or anxiety to ensure they receive allowances or rehabilitation. Thus, another important venue for future work involves detailed evaluation of physicians’ knowledge and general awareness of GD, and their specific reasons for assigning, or not assigning, GD diagnoses. Evidence shows that general practitioners rarely screen for or treat GD and often lack confidence in their knowledge or referral pathways [52]. Given that GD is seldom diagnosed in Finland compared to population estimates of gambling problems, more information is needed on healthcare practices related to GD, particularly the diagnostic process. Moreover, future research should combine data on received diagnoses with data on received allowances or rehabilitation to gain knowledge on which specific diagnoses form the basis for receiving such benefits.

While many individuals who would likely fulfil the diagnostic criteria for GD remain undiagnosed, those who are diagnosed usually have severe and complex problems. The negative consequences of GD, such as excessive preoccupation, guilt, and interpersonal strain, often require multiprofessional collaboration between healthcare and social services [53]. Clinical practices related to setting various diagnoses may have changed and developed over the years, resulting in differences among physicians and between various regions and units’ *modus operandi* relating to GD. The RPHC and CRHC registers covered the years 1996–2024 and 2011–2024, respectively, slightly complicating comparability. Despite these limitations, the Finnish health registers have good validity [54]. Previous register-based studies on GD comorbidity have covered only outpatient and inpatient specialised health care [10]– [22, 37], while recent Finnish register studies on GD have relied on aggregated data [9, 21]. Our study covered also primary health care visits and focused on longitudinal trajectories between GD and comorbid psychiatric conditions across nearly three decades.

## Conclusions

We have provided a population-wide description of the comorbid and temporal trajectories of GD and other psychiatric disorders among males and females. Our findings help social and health care professionals become more aware of comorbidities when working with affected individuals. To take adequate policy measures against GD, it is crucial to thoroughly model diagnosis occurrence, determinants and temporal trajectories in the healthcare system.

Ultimately, both GD and its comorbid disorders need to be recognized, diagnosed, and treated regardless of their temporal and causal orders. Better screening for GD among at-risk individuals is urgently needed, particularly among those seeking treatment for other psychiatric symptoms. Only one third of GD diagnoses were defined as primary, indicating that GD was typically identified during an evaluation focused on other disorders, highlighting the need for efficient *simultaneous treatment* of GD and any co-occurring psychiatric condition. Finally, both GD and internalizing disorders, including mood and anxiety disorders, are strongly linked with suicidal behaviour [55]– [56]. Systematic suicide-risk assessment is therefore essential in the context of GD and its comorbid conditions.

## Supplementary Information

Below is the link to the electronic supplementary material.


Supplementary Material 1


## Data Availability

A permission (statement: THL/4629/6.02.00/2024) to use the data were received from THL. The data are stored, used, and destroyed in accordance with the instructions and policies of THL. The individual level data were processed by a researcher, who had received access rights from THL to confidential datasets.These health register data are accessible only via permission from the Finnish Institute for Health and Welfare.

## Footnotes

1 The ICD-10 code F63.0 refers to *pathological gambling*. However, the more recent term GD is used in this paper due to the changes in the revised edition of the ICD (11th ed.; ICD-11, World Health Organization, 2019). In the ICD-11, the diagnostic term pathological gambling was changed to GD and placed under the category of Substance Use and Related Disorders.

2 Long-term GD diagnosis refers to a persistent or chronic classification of GD that indicates ongoing treatment needs or continued relevance to the patient’s health and well-being, even after the acute phase has passed. Such diagnoses are marked as persistent in-patient records, appear on the long-term diagnosis list compiled by the national data service, and remain there until explicitly marked as ended.

3 Data is not included from the Care Register for Social Welfare (CRSW), which contains information from social welfare institutions such as disability- and nursing homes, inpatient substance use care, and other 24/7 sheltered accommodations. However, the share of GD diagnoses registered solely in the CRSW is 2.99%, while the RPHC and CRHC contain 97.01% of all GD diagnoses (as of December 2024). Thus, the results would not have been significantly affected by including data from the CRSW.

4 See Table 2 for details, and the supplementary materials for a mathematical equation of the model.

5 Note that these data were sampled later and extend to 2025 (specifically October 5, 2025), while the main analysis extends to 2024.
