# Supplementary material for: Occurrence and trajectories of psychiatric disorders in adults with gambling disorder: a longitudinal register-based study of the Finnish population
Source: Soc Psychiatry Psychiatr Epidemiol. 2026 Feb 13;61(8):1499–511. doi: 10.1007/s00127-025-03034-8 (PMC13522059; doi:10.1007/s00127-025-03034-8)
Supplement: Supplementary file 1 [file 127_2025_3034_MOESM1_ESM.docx]

**Occurrence and trajectories of psychiatric disorders in adults with gambling disorder: a longitudinal study based on health registers covering the Finnish population**

**Supplementary Materials**

**Linear Mixed Model equation (centered parameterization)**

Difference in days

*µ = αj[i]* + *β*_1,m_*_[i]_* + *β*_2_Sex + *β*_3_Diagnosis_status + *β*_4_Register_source + *β*_5,m_*_[i]_*×Sex

, for Individual ID j = 1,*...*,J

The terms **j[i]** and **m[i]** denote the individual **j** and the ICD category level **m** the observation **i** belongs to. Thus, *β*_1,m_*_[i]_* denotes the estimated parameters for the dummy variables representing the levels of ICD category.

The lmer-syntax of the model was the following:

*lmer(Difference_in_days ~ ICD_category*Sex + GD_diagnosis_status + Register_source + (1 | Individual_ID))*

where *Sex, GD_diagnosis_status* and *Register_source* were dichotomous predictor variables, and *ICD_category* categorical with 12 levels. Treatment contrasts were used; thus *ICD_category* was transformed into 11 dummy variables with “Gambling disorder” representing the reference level.

**Supplementary figures**

Figures S1 – S4 depict the difference in time between receiving a gambling disorder (GD) diagnosis and a comorbid F-diagnosis, separately for males and females, as well as GD diagnosis status (primary diagnosis vs. secondary diagnosis [figure S1]; long-term vs. not long term [figure S2]), register source (figure S3); restricting the PHCR data range to begin from 2011, thus matching both registers’ coverage (figure S4); and for more detailed F-diagnosis categories (figure S5). In all figures, black/grey data points represent individual observations of the onsets of different comorbid diagnoses. Random vertical jitter is added to data points. The means for male (blue points) and female (red points) calculated from raw data. In figure S4, mean- and median values are calculated separately for comorbid diagnoses given before and after the GD diagnosis. The error bars are 95% confidence intervals.

**Figure S1**

**Figure S2**

**Figure S3**

*Note.* The Care register for health care (CRHC) covers the years 2011–2024, while the Primary health care register (PHCR) covers the years 1996–2024. The range of possible values for years before/after "Gambling disorder" diagnosis is [–13, 28] for CRHC and [-28, 28] for PHCR. Thus, the data is necessarily positively skewed for CRHC, and the sex-wise means are slightly positively biased. The results (figure 1A and 1B in the main text) are, however, essentially the same when restricting the PHCR data range to begin from 2011, thus matching both registers’ coverage (see figure S4, below).

**Figure S4**

*Note.* Data is restricted to 2011-2024, which guarantees identical data ranges for both health registers. Black/grey data points represent individual observations of the onsets of different comorbid diagnoses. Random vertical jitter is added to data points. **A (top panel):** The means for male (blue points) and female (red points) calculated from raw data. **B (bottom panel):** The means (and medians) calculated separately for values above (black) and below (dark blue) zero, with zero-values excluded from the data. The error bars are 95% confidence intervals.

**Figure S5**

**Table S1.** Diagnosis counts for 1st through 15th diagnosis.

|  | **Diagnosis number (1^st^ through 15^th^)** | | | | | | | | | | | | | | |  |
| --- | --- | --- | --- | --- | --- | --- | --- | --- | --- | --- | --- | --- | --- | --- | --- | --- |
| **F-diagnosis category** | **1st** | **2nd** | **3rd** | **4th** | **5th** | **6th** | **7th** | **8th** | **9th** | **10th** | **11th** | **12th** | **13th** | **14th** | **15th** | **Total** |
| **Gambling disorder** | **1572** | **764** | **591** | **392** | **307** | **271** | **224** | **154** | **142** | **116** | **116** | **88** | **69** | **47** | **46** | **5172** |
| **Mood disorders** | **1243** | **1559** | **1389** | **1298** | **1121** | **957** | **857** | **749** | **660** | **590** | **478** | **394** | **343** | **290** | **251** | **13493** |
| Unipolar depression  incl. dysthymia | 1175 | 1412 | 1203 | 1090 | 900 | 750 | 653 | 548 | 469 | 414 | 332 | 268 | 225 | 187 | 151 | 10518 |
| Bipolar affective  disorder | 48 | 107 | 138 | 177 | 190 | 186 | 193 | 175 | 172 | 166 | 131 | 119 | 111 | 96 | 90 | 2639 |
| Other / unspecific  mood disorders | 20 | 40 | 48 | 31 | 31 | 21 | 11 | 26 | 19 | 10 | 15 | 7 | 7 | 7 | 10 | 336 |
| **Anxiety disorders** | **971** | **1026** | **916** | **821** | **705** | **646** | **503** | **467** | **354** | **291** | **248** | **240** | **177** | **172** | **136** | **8518** |
| Phobic anxiety  disorders | 562 | 695 | 602 | 554 | 477 | 447 | 370 | 316 | 253 | 196 | 182 | 154 | 114 | 111 | 79 | 5645 |
| Adjustment and  stress-related  disorders | 320 | 265 | 251 | 220 | 175 | 140 | 86 | 115 | 72 | 67 | 47 | 61 | 46 | 49 | 38 | 2170 |
| Obsessive-  compulsive  disorder | 28 | 34 | 41 | 30 | 22 | 48 | 27 | 20 | 16 | 19 | 8 | 14 | 8 | 5 | 11 | 380 |
| Somatoform disorders | 55 | 29 | 21 | 16 | 29 | 10 | 19 | 15 | 11 | 9 | 9 | 11 | 9 | 6 | 8 | 298 |
| Other anxiety  disorders | 6 | 3 | 1 | 1 | 2 | 1 | 1 | 1 | 2 | 0 | 2 | 0 | 0 | 1 | 0 | 25 |
| **Substance use disorders** | **330** | **388** | **439** | **423** | **417** | **391** | **404** | **348** | **313** | **302** | **275** | **248** | **229** | **209** | **196** | **6635** |
| Alcohol use disorder | 242 | 282 | 311 | 280 | 279 | 275 | 264 | 229 | 187 | 174 | 165 | 142 | 130 | 112 | 97 | 3988 |
| Other or multiple drug  use disorder(s) | 28 | 38 | 48 | 46 | 50 | 35 | 41 | 39 | 45 | 42 | 35 | 37 | 34 | 32 | 37 | 896 |
| Benzodiazepine /  hypnotics use disorder | 6 | 9 | 17 | 25 | 18 | 27 | 30 | 23 | 26 | 33 | 24 | 16 | 16 | 27 | 24 | 577 |
| Cannabis use disorder | 25 | 26 | 25 | 33 | 31 | 26 | 25 | 23 | 17 | 20 | 17 | 10 | 14 | 14 | 12 | 395 |
| Opioid use disorder | 4 | 7 | 14 | 9 | 10 | 5 | 16 | 13 | 12 | 10 | 15 | 22 | 18 | 15 | 16 | 345 |
| Tobacco use disorder | 19 | 18 | 18 | 23 | 16 | 15 | 18 | 12 | 9 | 10 | 9 | 8 | 6 | 3 | 3 | 220 |
| Cocaine / other  stimulant use disorder | 6 | 8 | 6 | 7 | 13 | 8 | 10 | 9 | 17 | 13 | 10 | 13 | 11 | 6 | 7 | 214 |
| **Schizophrenia spectrum disorders** | **215** | **213** | **202** | **211** | **178** | **175** | **166** | **141** | **122** | **126** | **112** | **87** | **96** | **77** | **62** | **2619** |
| Schizophrenia | 45 | 76 | 70 | 85 | 85 | 83 | 83 | 67 | 51 | 56 | 47 | 34 | 42 | 32 | 28 | 1050 |
| Other / unspecified  nonorganic  psychosis | 114 | 63 | 60 | 62 | 32 | 45 | 26 | 23 | 21 | 20 | 27 | 13 | 16 | 10 | 8 | 599 |
| Schizoaffective  disorders | 8 | 11 | 19 | 17 | 26 | 23 | 24 | 23 | 26 | 27 | 24 | 24 | 27 | 25 | 16 | 458 |
| Acute psychotic  disorders | 34 | 48 | 29 | 24 | 18 | 18 | 20 | 12 | 12 | 14 | 8 | 11 | 7 | 9 | 5 | 304 |
| Persistent delusional  disorders | 9 | 10 | 19 | 18 | 10 | 5 | 9 | 8 | 8 | 6 | 4 | 3 | 3 | 0 | 3 | 142 |
| Schizotypal disorder | 5 | 5 | 5 | 4 | 7 | 1 | 4 | 8 | 4 | 3 | 2 | 2 | 1 | 1 | 2 | 65 |
| Other schizophrenia  spectrum disorder | 0 | 0 | 0 | 1 | 0 | 0 | 0 | 0 | 0 | 0 | 0 | 0 | 0 | 0 | 0 | 1 |
| **Personality disorders** | **83** | **135** | **154** | **157** | **175** | **152** | **126** | **144** | **162** | **109** | **93** | **101** | **82** | **68** | **52** | **2140** |
| **Behavioural syndromes** | **211** | **199** | **200** | **159** | **153** | **131** | **117** | **103** | **77** | **74** | **60** | **46** | **45** | **42** | **32** | **1879** |
| Non-organic sleep  disorders | 170 | 156 | 162 | 129 | 124 | 107 | 96 | 82 | 60 | 64 | 43 | 38 | 35 | 34 | 25 | 1501 |
| Eating disorders | 19 | 21 | 13 | 14 | 14 | 13 | 9 | 9 | 8 | 5 | 8 | 2 | 3 | 4 | 1 | 181 |
| Sexual dysfunction | 17 | 20 | 22 | 12 | 14 | 11 | 9 | 12 | 9 | 5 | 8 | 5 | 7 | 3 | 5 | 172 |
| Other behavioral  syndromes | 5 | 2 | 3 | 4 | 1 | 0 | 3 | 0 | 0 | 0 | 1 | 1 | 0 | 1 | 1 | 25 |
| **Behav. and emot. disorders in childhood and adolescence** | **317** | **242** | **177** | **136** | **101** | **84** | **65** | **52** | **46** | **41** | **29** | **22** | **24** | **14** | **14** | **1440** |
| ADHD spectrum | 96 | 86 | 74 | 67 | 54 | 51 | 42 | 39 | 35 | 32 | 27 | 14 | 20 | 10 | 14 | 727 |
| Other behav. and  emot. disorders in... | 126 | 92 | 53 | 42 | 25 | 21 | 12 | 11 | 7 | 7 | 1 | 7 | 4 | 4 | 0 | 418 |
| Conduct disorders  (incl. oppositional  defiant disorder) | 95 | 64 | 50 | 27 | 22 | 12 | 11 | 2 | 4 | 2 | 1 | 1 | 0 | 0 | 0 | 295 |
| **Disorders of psychol. development** | **174** | **87** | **70** | **41** | **42** | **29** | **29** | **20** | **24** | **17** | **12** | **14** | **6** | **7** | **8** | **600** |
| Other developmental  disorders | 163 | 75 | 52 | 25 | 19 | 19 | 20 | 10 | 8 | 7 | 9 | 8 | 3 | 5 | 4 | 442 |
| Autism spectrum  disorder | 11 | 12 | 18 | 16 | 23 | 10 | 9 | 10 | 16 | 10 | 3 | 6 | 3 | 2 | 4 | 158 |
| **Organic mental disorder** | **14** | **35** | **27** | **34** | **32** | **18** | **23** | **22** | **13** | **8** | **10** | **7** | **3** | **5** | **4** | **299** |
| **Unspecified mental disorder** | **36** | **45** | **34** | **21** | **22** | **18** | **16** | **12** | **11** | **8** | **6** | **7** | **4** | **2** | **6** | **288** |
| **Intellectual disability** | **6** | **9** | **12** | **9** | **12** | **9** | **13** | **6** | **10** | **1** | **2** | **5** | **4** | **3** | **3** | **112** |

*Note*. Diagnosis numbers > 15 are omitted but counted towards “Total”. Date of sampling: 1.10.2024.

Table 2 presents the number of individuals who were given a specific diagnosis as their *n*th diagnosis (n = 1 ... 15). Data is shown only up to the 15^th^ diagnosis for simplicity and presented in decreasing order of total diagnosis counts.

**Table S2.** Diagnosis occurrence counts for GD vs. psychiatric controls across all ICD categories. Counts are sorted from highest to lowest for GD counts.

|  |  | **Gambling disorder** | | **Psychiatric controls** | |
| --- | --- | --- | --- | --- | --- |
| **F-codes** | **ICD category** | **Count** | **Count / Total** | **Count [99% CI]** | **Count / Total** |
| 32–34 | Unipolar depression, incl. dysthymia | 12159 | 0.271 | 5428 [5131—5787] | 0.232 |
| 40–41 | Phobic anxiety disorders | 6865 | 0.153 | 3878 [3678—4100] | 0.166 |
| 10 | Alcohol use disorder | 4525 | 0.100 | 2117 [1862—2363] | 0.908 |
| 30–31 | Bipolar disorders | 2978 | 0.664 | 646 [499—786] | 0.277 |
| 43–44 | Adjustment and stress related disorders | 2661 | 0.593 | 2065 [1943—2210] | 0.886 |
| 60–69 | Personality disorders | 2340 | 0.521 | 617 [528—712] | 0.265 |
| 51 | Nonorganic sleep disorders | 1958 | 0.436 | 1933 [1819—2061] | 0.829 |
| 20 | Schizophrenia | 1129 | 0.252 | 440 [344—561] | 0.189 |
| 16, 18–19 | Other or multiple drug use disorder | 1038 | 0.231 | 513 [403—630] | 0.220 |
| 90.0, 90.8, 90.9, 98.8 | ADHD spectrum | 983 | 0.219 | 606 [540—671] | 0.260 |
| 28–29 | Other/unspecified nonorganic psychosis | 689 | 0.154 | 345 [300—393] | 0.148 |
| 13 | Benzodiazepine/hypnotics use disorder | 650 | 0.145 | 248 [188—315] | 0.106 |
| 80–83 | Other developmental disorders | 594 | 0.132 | 533 [457—616] | 0.229 |
| 93–95, 98 | Other behav. and emot. disorders in childhood and adolescence | 590 | 0.131 | 389 [325—443] | 0.167 |
| 25 | Schizoaffective disorders | 501 | 0.112 | 92 [57—133] | 0.039 |
| 12 | Cannabis use disorder | 486 | 0.108 | 194 [148—252] | 0.083 |
| 42 | Obsessive-compulsive disorder | 454 | 0.101 | 214 [165—267] | 0.092 |
| 90.1, 91–92 | Conduct disorder, incl. ODD | 440 | 0.098 | 210 [162—262] | 0.090 |
| 11 | Opioid use disorders | 423 | 0.094 | 308 [226—402] | 0.132 |
| 38–39 | Other/unspecified mood disorder | 411 | 0.092 | 159 [127—192] | 0.068 |
| 45 | Somatoform disorders | 339 | 0.075 | 434 [377—488] | 0.186 |
| 23 | Acute psychosis | 339 | 0.075 | 152 [110—195] | 0.065 |
| 99 | Unspecified mental disorder | 330 | 0.073 | 132 [103—161] | 0.057 |
| 00–09 | Organic mental disorder | 326 | 0.073 | 189 [146—237] | 0.081 |
| 14–15 | Cocaine/other stimulant use disorder | 281 | 0.063 | 148 [100—199] | 0.063 |
| 17 | Tobacco use disorder | 278 | 0.062 | 174 [138—210] | 0.075 |
| 52 | Sexual dysfunction | 223 | 0.050 | 435 [379—490] | 0.187 |
| 84 | Autism spectrum disorders | 217 | 0.048 | 164 [124—204] | 0.070 |
| 50 | Eating disorders | 215 | 0.048 | 108 [73—150] | 0.046 |
| 22 | Persistent delusional disorder | 155 | 0.035 | 80 [54—107] | 0.034 |
| 70–79 | Intellectual disability | 128 | 0.029 | 249 [186—314] | 0.107 |
| 21 | Schizotypal disorder | 70 | 0.015 | 31 [18—45] | 0.013 |
| 88–89 | Other disorders of psychological development | 38 | 0.008 | 31 [17—47] | 0.013 |
| 53.0, 53.1, 53.9, 54, 55, 59 | Other behavioral syndromes | 34 | 0.008 | 28 [15—43] | 0.012 |
| 48.0, 48.1, 48.8, 48.9 | Other anxiety disorders | 30 | 0.007 | 26 [14—38] | 0.011 |
| 24 | Other schizophrenia spectrum disorder | 1 | 0.000 | 0 [0—0] | 0.000 |
|  | **Total count** | **44876** |  | **23316** |  |

Note. Green cells indicate when the GD population parameter is outside the cluster-bootstrapped 99% confidence intervals for the psychiatric control group diagnosis counts, indicating statistical significance at approximately the 0.01 level. Thus green = statistically higher diagnosis occurrence count. N for both groups = 5935 (date of sampling: 4.10.2025).

**Table S3.** STROBE Statement checklist

|  | **Item No** | **Recommendation** | **Page No** |
| --- | --- | --- | --- |
| **Title and abstract** | 1 | (*a*) Indicate the study’s design with a commonly used term in the title or the abstract | 1 |
|  |  | (*b*) Provide in the abstract an informative and balanced summary of what was done and what was found | 4 |
| **Introduction** | | | |
| Background/rationale | 2 | Explain the scientific background and rationale for the investigation being reported | 5 |
| Objectives | 3 | State specific objectives, including any prespecified hypotheses | 7 |
| **Methods** | | | |
| Study design | 4 | Present key elements of study design early in the paper | 7 |
| Setting | 5 | Describe the setting, locations, and relevant dates, including periods of recruitment, exposure, follow-up, and data collection | 7-8 |
| Participants | 6 | (*a*) Give the eligibility criteria, and the sources and methods of selection of participants | 7-8 |
| Variables | 7 | Clearly define all outcomes, exposures, predictors, potential confounders, and effect modifiers. Give diagnostic criteria, if applicable | 7-10 |
| Data sources/ measurement | 8* | For each variable of interest, give sources of data and details of methods of assessment (measurement). Describe comparability of assessment methods if there is more than one group | 8 |
| Bias | 9 | Describe any efforts to address potential sources of bias | 9-10 |
| Study size | 10 | Explain how the study size was arrived at | 7 |
| Quantitative variables | 11 | Explain how quantitative variables were handled in the analyses. If applicable, describe which groupings were chosen and why | 7-9 |
| Statistical methods | 12 | (*a*) Describe all statistical methods, including those used to control for confounding | 9-10 |
|  |  | (*b*) Describe any methods used to examine subgroups and interactions | 9-10 |
|  |  | (*c*) Explain how missing data were addressed | 9-10 |
|  |  | (*d*) If applicable, describe analytical methods taking account of sampling strategy | 9-10 |
|  |  | (*e*) Describe any sensitivity analyses | 9-10 |
| **Results** | | | |
| Participants | 13* | (a) Report numbers of individuals at each stage of study—eg numbers potentially eligible, examined for eligibility, confirmed eligible, included in the study, completing follow-up, and analysed | 10 |
|  |  | (b) Give reasons for non-participation at each stage |  |
|  |  | (c) Consider use of a flow diagram |  |
| Descriptive data | 14* | (a) Give characteristics of study participants (eg demographic, clinical, social) and information on exposures and potential confounders | 8 |
|  |  | (b) Indicate number of participants with missing data for each variable of interest | 8 |
| Outcome data | 15* | Report numbers of outcome events or summary measures | 7-10 |
| Main results | 16 | (*a*) Give unadjusted estimates and, if applicable, confounder-adjusted estimates and their precision (eg, 95% confidence interval). Make clear which confounders were adjusted for and why they were included | 14-15 |
|  |  | (*b*) Report category boundaries when continuous variables were categorized | 14-15 |
|  |  | (*c*) If relevant, consider translating estimates of relative risk into absolute risk for a meaningful time period |  |
| Other analyses | 17 | Report other analyses done—eg analyses of subgroups and interactions, and sensitivity analyses | 16-18 |

**Table S3.** *Continued from previous page.*

| **Discussion** | | | |
| --- | --- | --- | --- |
| Key results | 18 | Summarise key results with reference to study objectives | 19 |
| Limitations | 19 | Discuss limitations of the study, taking into account sources of potential bias or imprecision. Discuss both direction and magnitude of any potential bias | 20-21 |
| Interpretation | 20 | Give a cautious overall interpretation of results considering objectives, limitations, multiplicity of analyses, results from similar studies, and other relevant evidence | 22 |
| Generalisability | 21 | Discuss the generalisability (external validity) of the study results | 19-22 |
| **Other information** | | | |
| Funding | 22 | Give the source of funding and the role of the funders for the present study and, if applicable, for the original study on which the present article is based | 22 |

**Table S4.** Year-wise GD diagnosis counts for both health registers

| **Year** | **Primary health care register** | **Care register for health care** |
| --- | --- | --- |
| 1996 | 35 | NA |
| 1997 | 15 | NA |
| 1998 | 56 | NA |
| 1999 | 48 | NA |
| 2000 | 47 | NA |
| 2001 | 68 | NA |
| 2002 | 48 | NA |
| 2003 | 46 | NA |
| 2004 | 55 | NA |
| 2005 | 54 | NA |
| 2006 | 71 | NA |
| 2007 | 68 | NA |
| 2008 | 73 | NA |
| 2009 | 58 | NA |
| 2010 | 92 | NA |
| 2011 | 79 | 47 |
| 2012 | 101 | 49 |
| 2013 | 92 | 51 |
| 2014 | 119 | 59 |
| 2015 | 109 | 92 |
| 2016 | 95 | 113 |
| 2017 | 144 | 142 |
| 2018 | 182 | 185 |
| 2019 | 179 | 193 |
| 2020 | 174 | 266 |
| 2021 | 182 | 320 |
| 2022 | 123 | 362 |
| 2023 | 123 | 403 |
| 2024 | 85 | 269 |
| **TOTAL** | **2621** | **2551** |

*Note*. Diagnosis counts include individuals who receive their initial GD diagnosis on a given year. The inclusion of private service providers in the care register for health care data collection since 2020 has significantly increased the total number of visits. Date of sampling: 1.10.2024.
